# Therapeutic Success in Swiss COPD Patients Receiving Dual Bronchodilation Therapy as COPD Maintenance Treatment

**DOI:** 10.3390/clinpract12010006

**Published:** 2022-01-07

**Authors:** Marc Spielmanns, Sebastian Schildge, Jens Peter Diedrich, Arschang Valipour

**Affiliations:** 1 Pulmonary Medicine, Zuercher RehaZentren Klinik Wald, 8636 Wald, Switzerland; 2 Department of Pneumology, Faculty of Health, University of Witten/Herdecke, 58455 Witten, Germany; 3 Medical Affairs, Boehringer Ingelheim, 4002 Basel, Switzerland; sebastian.schildge@boehringer-ingelheim.com (S.S.); jens.diedrich@boehringer-ingelheim.com (J.P.D.); 4 Department of Respiratory and Critical Care Medicine, Karl-Landsteiner-Institute for Lung Research and Pulmonary Oncology, Klinik Floridsdorf, 1210 Vienna, Austria; arschang.valipour@gesundheitsverbund.at

**Keywords:** chronic obstructive pulmonary disease (COPD), Switzerland, Clinical COPD Questionnaire (CCQ), physicians global evaluation (PGE) score

## Abstract

Health-related quality of life (HRQoL) in patients with moderate to severe chronic obstructive pulmonary disease (COPD) is often reduced by high symptom burden and frequent exacerbations. So far, data on therapeutic success in Swiss COPD patients receiving dual bronchodilation therapy as COPD maintenance treatment are limited. Data from a recently published, non-interventional study on clinical benefit after the start of combined tiotropium–olodaterol treatment were analyzed focusing on Swiss patients compared to the overall cohort including patients from various European countries. Demographic data on the changes in Clinical COPD Questionnaire (CCQ) for the assessment of HRQoL in correlation to symptoms and the number of exacerbations, as well as physician’s global assessment (PGE), were evaluated 6 weeks after treatment start. In Switzerland (*n* = 61), significantly more patients had comorbidities and exacerbations but showed less symptoms compared to the overall cohort (*n* = 4639). HRQoL improved in both cohorts, with a negative correlation to symptom burden and number of exacerbations in the overall cohort. PGE scores improved after 6 weeks with a better general condition at baseline in Swiss patients (PGE score 4/5: 68.9% [Swiss cohort] vs. 49.0% [overall cohort]. Despite significant differences regarding the presence of symptoms and exacerbations, therapeutic success was similar in both patient groups. Highly symptomatic patients benefited mostly from tiotropium–olodaterol treatment.

## 1. Introduction

Chronic obstructive pulmonary disease (COPD) is a common, treatable disease characterized by persistent respiratory symptoms, airflow limitation, and recurrent acute exacerbations [1]. These symptoms, as well as the effects of concomitant diseases such as cor pulmonale or pulmonary hypertension, significantly limit patients in their daily activities and reduce their health-related quality of life (HRQoL) [2,3,4]. Frequent hospitalizations and increased mortality are further complications in COPD, which also have a notable economic and social impact [1,5,6]. 

Key treatment in COPD includes maintenance bronchodilator therapy with oral inhaled long-acting muscarinic antagonists (LAMAs) and long-acting β_2_-agonists (LABA) to reduce symptom burdens as well as frequency and severity of exacerbations, and to improve patients´ quality of life and exercise capacity [1]. For patients with moderate to very severe COPD, combined therapy with LAMA + LABA is recommended [1,7,8,9]. Various studies showed that the dual strategy of bronchodilatation provides an economically sensible way to gain clinical benefit and enhanced patient reported outcomes [10,11,12].

The Spiolto Respimat^®^ soft mist inhaler combines two well-known bronchodilators, tiotropium, a well-established LAMA, and olodaterol, a LABA with rapid onset of efficacy [13,14]. Clinical studies regarding COPD treatment with the Spiolto Respimat^®^ inhaler have shown significant improvements in lung function, respiratory symptoms, HRQoL, and exercise capacity [15,16,17,18,19]. In a recently published, real-world, observational study of Valipour et al., treatment with tiotropium–olodaterol via a Spiolto Respimat^®^ inhaler confirmed those results as an improvement in self-reported HRQoL, as assessed by the Clinical COPD Questionnaire (CCQ) [20]. 

The CCQ is a well-known, easy-to-use, and multi-lingual validated tool which can assess the clinical impact of COPD treatment on patients´ specific needs and their HRQoL [21]. Using the three domains, symptoms, functional state, and mental state, the CCQ reliably reflects the individual situation and symptom burden of COPD patients [21].

In most of the patients, disease control is achievable with dual bronchodilator therapy [22], but in the literature, quite different country-specific responses to therapeutic interventions were observed in COPD patients [23]. Up to now, data from COPD patients receiving medical therapy in Swiss clinical practice are limited [24]. In the current study, we analysed data obtained during the OTIVACTO study of Valipour et al. [20] which focusses on the results of the Swiss patients. Results of the Swiss cohort were compared to the patients from the other participating countries for a better understanding of the clinical outcomes in the subgroup of Swiss COPD patients treated with inhaled tiotropium–olodaterol in comparison to the whole OTIVACTO data.

## 2. Materials and Methods

### 2.1. Study Design

This non-interventional study was an open-label, self-controlled, single-arm, observational study enrolling male and female patients aged ≥40 years with confirmed diagnosis of COPD receiving tiotropium–olodaterol delivered via Spiolto Respimat^®^ soft mist inhaler. A detailed description of the study design has been published previously by Valipour et al. [20]. Briefly, GOLD patients from group B, C, or D (GOLD COPD Strategy Document 2018) [1] were consecutively enrolled upon the decision of their treating physician and in accordance with approved summary of product characteristics (SmPC) [25]. Patients from Switzerland, Bulgaria, Czech Republic, Hungary, Israel, Lithuania, Poland, Romania, Russia, Slovenia, and Ukraine participated in the study. Patients were followed over an observational period of approximately 6 weeks, which represents the average time between two medical consultations. The primary endpoint was the therapeutic success at visit 2 which was defined as a 0.4-point decrease in the CCQ score between baseline visit (visit 1) and visit 2 approximately 6 weeks after treatment started. The general condition of the patients was assessed by the Physician´s Global Evaluation (PGE) score at baseline and at visit 2. Further assessments comprised demographic data, disease characteristics, concomitant diseases and medication, smoking status, exacerbations, patients´ satisfaction and willingness to continue treatment after study end, use of rescue medication (e.g., short-acting bronchodilators), and safety [20].

### 2.2. Ethical Considerations

The study was approved by country-specific independent ethic committees for each participating country and was carried out in accordance with the principles of the declaration of Helsinki. Identifiers: BASEC 2018-01259 (Switzerland); NCT03663569 (clinicaltrials.gov). Written informed consent was obtained from all individual participants prior to study enrollment. 

### 2.3. Statistical Analysis

Data were analyzed using SAS version 9.4 (Cary, NC, USA). All data were expressed as mean ± SD or as *n* and %.

Therapeutic success was defined as a change of −0.4 points in the CCQ score between visit 1 and visit 2. The correlation between the change in CCQ score and certain baseline characteristics were analysed by means of Pearson correlation coefficients. In this analysis, we extracted the data of the Swiss patient group and compared the results to the rest of the cohort, including patients from all other participating countries.

For comparison of continuous variables, the Wilcoxon rank sum test (Mann–Whitney U test) or Kruskal–Wallis test was used. Categorical variables were compared using the χ² test, and if the χ² test was not valid, the Fisher’s exact test was used. All analyses were performed in descriptive manner and *p*-values were interpreted nominally. A *p*-value of *p* < 0.05 was considered as significant. Missing observations were not considered for analysis.

## 3. Results

### 3.1. Patient Demographics and Baseline Characteristics

The total cohort consists of 4700 patients, including 61 patients from Switzerland. Table 1 gives an overview about patients´ demographics and baseline characteristics. Significant differences between the Swiss cohort and the patients from other countries were observed in terms of gender distribution, disease characteristics, concomitant diseases and medication, and exacerbation history. In contrast to the other countries, in Switzerland, both genders were represented almost equally, more patients had a Modified British Medical Research Council (mMRC) Grade of 0–1, and the proportion of patients belonging to GOLD group C was larger. Additionally, in the Swiss cohort, a higher proportion of patients had ≥1 exacerbation in the last 12 months prior to study inclusion, and the mean number of exacerbations was higher than in the total cohort excluding Switzerland. Furthermore, Swiss patients had a higher proportion of concomitant diseases and concomitant medication compared with the larger cohort. Most frequently reported comorbidities of the Swiss cohort were (multiple answers possible) cardiovascular diseases (*n* = 38), metabolic/endocrinological diseases (*n* = 29), gastrointestinal/hepatobiliary diseases (*n* = 22, including 17 patients with gastroesophageal reflux disease (GERD)), and psychiatric diseases (*n* = 14).

### 3.2. Change in CCQ-Score According to Baseline mMRC Score

**Figure 1:**
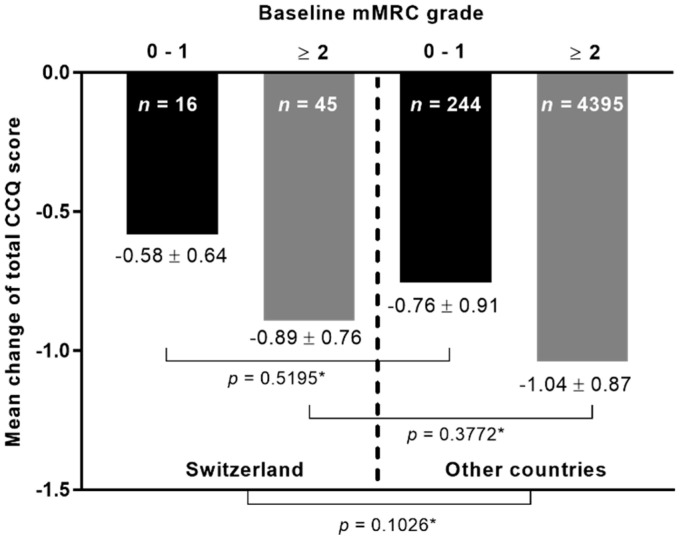
The mean change in the total CCQ score at visit 2 according to baseline mMRC grade. *n*: Number of patients. * Kruskal–Wallis test.

The mean change in the total CCQ score following therapy with tiotropium–olodaterol was stratified according to the mMRC grade at baseline and study cohort (Figure 1). Results indicated no significant differences in the mean change between patients from Switzerland in comparison to patients from the other countries, irrespective of the degree of breathlessness at baseline (Figure 1). 

### 3.3. Change in CCQ-Score According to Number of Exacerbations in the Last 12 Months

**Figure 2:**
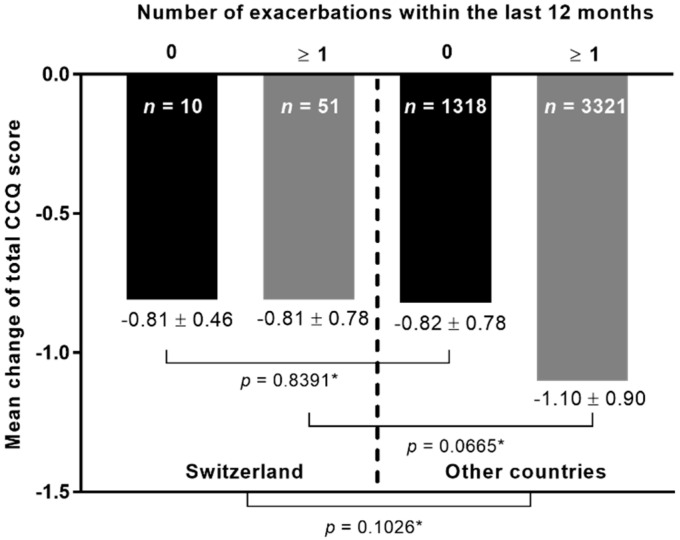
The mean change in the total CCQ score at visit 2 according to number of exacerbations within the last 12 months before study inclusion. *n*: number of patients. * Kruskal–Wallis test.

The mean change in the total CCQ score was additionally compared between the patients from Switzerland and the patients from the other countries with respect to the number of exacerbations within the last 12 months (Figure 2). Results showed no significant differences between both cohorts in general (*p*-value = 0.1026). Additionally, there were no statistically significant differences between patients from both cohorts with no exacerbations in the last 12 months prior study inclusion, as well as between patients from both cohorts with ≥1 exacerbations.

### 3.4. Relationships between Changes in CCQ Score, mMRC Score and Exacerbation Rate

**Figure 3:**
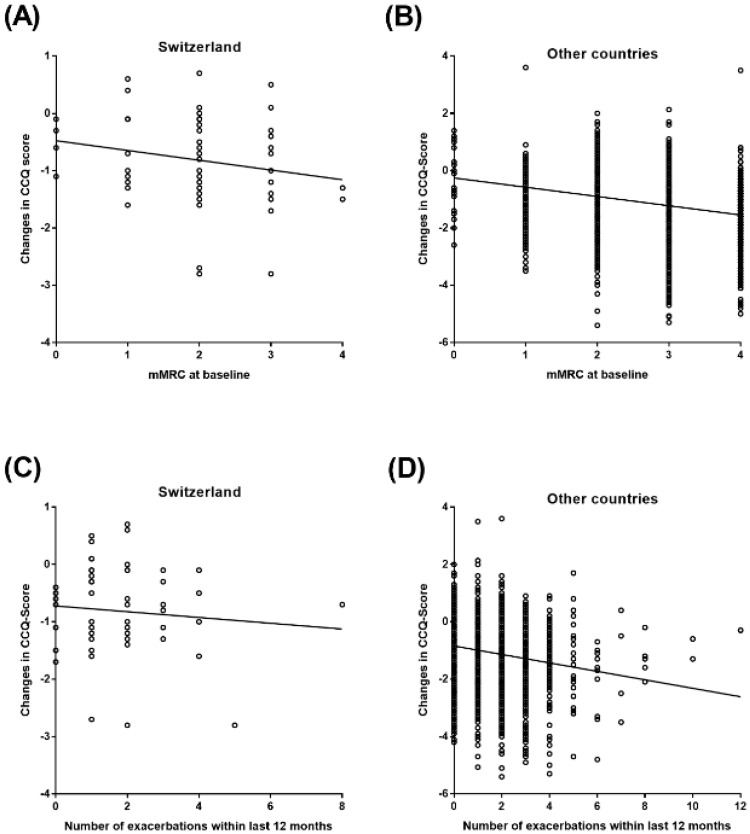
Pearson Correlation between CCQ score and mMRC at baseline and number of exacerbations within the last 12 months. (**A**) Pearson Correlation between CCQ score and mMRC at baseline in Switzerland; (**B**) Pearson Correlation between CCQ score and mMRC at baseline in other countries; (**C**) Pearson Correlation between CCQ score and number of exacerbations within the last 12 months in Switzerland; (**D**) Pearson Correlation between CCQ score and number of exacerbations within the last 12 months in other countries.

The results of the Pearson correlation analysis between the changes in the total CCQ score, the mMRC score, and exacerbation frequency within the last 12 months prior to study inclusion in patients from Switzerland and from the other countries are shown in Figure 3 and Table 2. Results indicate a significant negative correlation between the mMRC score at baseline and the changes in total CCQ score in the larger cohort (*p*-value < 0.0001) but not in Switzerland, independently (*p*-value = 0.1068). A similar result was observed for the correlation between the number of exacerbations and the CCQ score change with a significant negative correlation in the other countries (*p*-value < 0.0001). Again, in the Swiss cohort, no significant correlation between both variables was observed (*p*-value = 0.4543). 

### 3.5. PGE Score Distribution at Visit 1 and Visit 2

**Figure 4:**
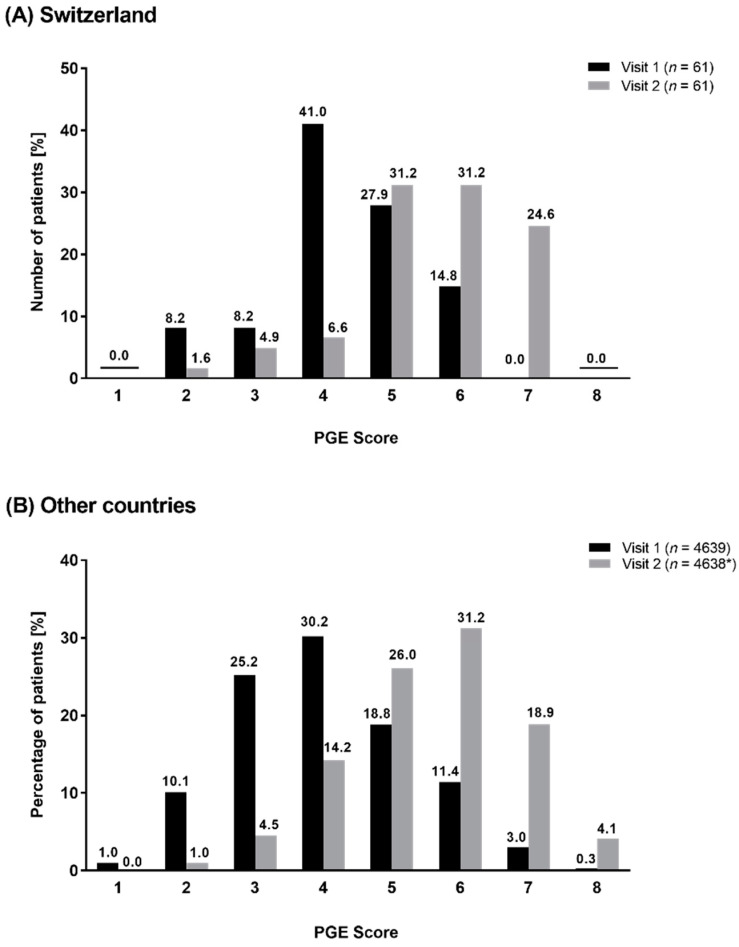
Distribution of PGE Score at visit 1 and at visit 2 in Switzerland (**A**) and in other countries (**B**). * one patient was excluded.

At baseline, Swiss patients mostly documented a satisfactory-to-good general condition since most of the patients had a PGE score of 4 (41.0%, *n* = 25) or 5 (27.9%, *n* = 17, Figure 4A). This represented a distribution differently to the other countries, where most of the patients had a PGE of 3 (25.2%, *n* = 1171) or 4 (30.2%, *n* = 1403, Figure 4B). After 6 weeks of treatment, the PGE score improved in both cohorts, with a majority of patients in both cohorts reporting PGE scores of 5 (Switzerland: 31.2%, *n* = 19; other countries: 26.0%, *n* = 1208) or 6 (Switzerland: 31.2%, *n* = 19; other countries: 31.2%, *n* = 1449, Figure 4A,B). Details regarding the PGE scores in both cohorts are shown in Figure 4.

## 4. Discussion

In the current analysis, HRQoL in COPD patients was markedly improved after onset of dual therapy with tiotropium–olodaterol without significant differences between patients from Switzerland and the pooled cohort of patients from the other countries. This is an important outcome as the results indicate that the LAMA/LABA combination tiotropium–olodaterol adequately reduces respiratory symptoms in a wide range of different patient populations. In a real-world environment, COPD patients are faced with quite different conditions in terms of health care systems, treatment management, and socio-economic circumstances. It is widely considered that such diversities can influence patients´ responsiveness to therapeutic approaches, although the reasons are still unknown [26]. However, in the current study, the improvement in HRQoL measured by the mean change in CCQ was similar in patients from both cohorts. The same results were also observed with consideration of the symptomatic burden represented by the mMRC score and the number of exacerbations in the last 12 months. In both cohorts, the mean change in CCQ was higher in patients with a mMRC score of ≥2, suggesting that those patients are most likely to benefit even more from the inhaler treatment with tiotropium–olodaterol. In the overall cohort, a weak, inverse correlation between the mean change in CCQ and mMRC grade strengthens this observation. Other publications have also shown that symptomatic patients exhibited the most prominent clinical benefit from the dual therapy with tiotropium–olodaterol [24,27,28]. We found no correlation between exacerbations and changes in the CCQ score in the Swiss group despite most of the patients belonging to GOLD group C and D, characterized by a high risk for exacerbations. In contrast, a statistically significant—albeit weak—inverse correlation was found in the overall cohort in this context. The differences between the Swiss and non-Swiss cohort reported here may, however, be largely driven by the obvious differences in sample size. Nevertheless, an improvement in HRQoL was observed in all patients from both cohorts, which confirms results from previous studies of inhaled bronchodilator therapy in COPD [15,20,29].

Although no differences regarding treatment response were observed between patients from Switzerland and patients from the other participating countries, some varieties between both cohorts were still present. In Switzerland, the proportion of patients belonging to GOLD group C was significantly higher in comparison to the other countries. This suggested that, in Switzerland, more patients suffer from COPD clinically present with less symptoms but with a higher risk for exacerbations in contrast to patients from the other countries, where most of the patients belonged to GOLD group B or D and, therefore, with an overall higher symptom burden. The high proportion of Swiss patients in GOLD group C and D was in line with data regarding exacerbation rates in the Swiss cohort. In the current study, a higher percentage of Swiss patients had ≥1 exacerbations within the previous 12 months in comparison to the other countries. Additionally, the number of exacerbations per patient was significantly higher in the Swiss group than in the overall cohort. However, reasons for the higher proportion of exacerbators in the Swiss cohort remain unclear. Acute exacerbations in COPD are mainly caused by respiratory infections (50% to 70%) [30] but, nevertheless, 30% are of unknown etiology [31]. A study by Buess et al. (2017) investigated acute exacerbations in Swiss COPD patients [32]. In their analysis, the percentage of smokers was significantly higher in the Swiss cohort than in the cohort including other European countries and a smaller proportion of Swiss patients received short-acting β_2_-agonists before exacerbation-related hospitalization, which may partially explain differences between countries. 

The general condition at baseline was slightly better in the Swiss than in the overall cohort which could be reasoned by the higher proportion of GOLD group C patients in Switzerland, since those patients experience less COPD related symptoms. However, the share of patients with concomitant diseases was significantly higher in the Swiss cohort than in the overall cohort. This seems to be contradictory to their good general condition. A possible explanation can be the well-established healthcare system in Switzerland enabling an excellent medical service to Swiss citizens [33]. Most of the Swiss COPD patients might receive adequate medical attention. Consequently, they reported a good general condition despite a higher prevalence of concomitant diseases compared with patients from the other participating countries. At visit 2, an improvement in the PGE score was observed in both cohorts without obvious differences between them. Those results confirm that bronchodilators are an effective treatment in most of the COPD patients, leading to a reduction in the symptom burden and achievement of an improved general condition [15,17,18,20,27]. Nevertheless, the improvement of the general condition appears to be slightly smaller in magnitude in Swiss patients than in patients from the other countries since Swiss patients started with a better PGE score at baseline and achieved mostly a similar score as the overall cohort. In the overall cohort, most of the patients belonged to GOLD group B or D and, thus, in countries other than Switzerland, a greater share of patients was highly symptomatic which might explain the pronounced improvement in the PGE score in those patients after the start of treatment. However, in the Swiss cohort, the share of patients with comorbidities was significantly higher (*p*-value: 0.0017) than in the overall cohort, and a considerable proportion suffered from depression and/or GERD at baseline. An improvement in COPD symptoms is often accompanied by a reduced fear of exacerbations which in turn has a positive effect on the patient´s mind. In patients suffering from GERD, stressful circumstances as being afraid of exacerbation can also worsen the symptoms [34]. An adequate symptom control by combined LABA/LAMA therapy decreases the stress level in COPD patients, but it can only be speculated whether this represents another reason for the improvement in PGE score in the Swiss cohort.

Our analysis has some limitations. It was performed as a post-hoc analysis to generate data on COPD patients from Switzerland and to compare those results to a large international patient cohort. The absolute number of Swiss patients was indeed small, and therefore, the results may not entirely reflect the differences between Switzerland and the other participating countries. However, since patients were included from the whole country without regional restrictions, the Swiss cohort can be considered as representative for the total Swiss COPD patient cohort. Further, data were generated during a non-interventional, observational study without having a comparison group to detect treatment efficacy in COPD as the primary effectiveness outcome in the overall population. It is discussed in the literature that real-life data (RLD) are more relevant for routine practice since they represent a real-world setting rather than an experimental setting. Usually, RLD study groups are heterogenous, involving many practitioners, and represent many alternative interventions. Another limitation represents the observational period which was rather short with 6 weeks after treatment onset. Therefore, long-lasting therapeutic effects could not be evaluated. Nevertheless, the current study was performed on a large, representative sample size in a real-world setting, including different kinds of healthcare systems and various COPD stages with mixed comorbidities and different previous treatment regimens. Therefore, results can be considered as valuable data comprehensively reflecting clinical practice in Switzerland in comparison to other European countries.

To summarize, the current analysis revealed no significant differences between COPD patients from Switzerland and from the other participating countries in treatment response based on an improvement in HRQoL, despite different healthcare systems and differences in the socio-economic environment. In both cohorts, the response to combined tiotropium–olodaterol treatment was good, and the general condition of the patients improved during the observational period. Our findings confirm the results of clinical trials, and other non-interventional, observational studies that combined tiotropium–olodaterol therapy represents an appropriate treatment option in COPD patients of GOLD group B, C, and D and should, thus, be administered in accordance with GOLD recommendations as the primary maintenance therapy in eligible patients.

## Figures and Tables

**Table 1:** Demographic data and baseline characteristics of the Swiss cohort and the other countries.

Demographic Data and Baseline Characteristics	Switzerland (*n* = 61)	Other Countries (*n* = 4639)	*p*-Value
Age at registration (years)			
*n*	61	4639	0.9247
Mean	65.15	65.34	
SD	11.50	9.31	
Min	42.00	40.00	
Median	66.00	66.00	
Max	87.00	93.00	
Age at registration (*n* (%))			
≤65 years	30 (49.18)	2294 (49.45)	0.9666
>65 years	31 (50.82)	2345 (50.55)	
Gender [*n* (%)]			
Male	32 (52.46)	3256 (70.19)	0.0027
Female	29 (47.54)	1383 (29.81)	
COPD degree of severity (spirometric) (*n* (%))			
1	3 (4.92)	150 (3.23)	0.6674
2	31 (50.82)	2578 (55.57)	
3	24 (39.34)	1547 (33.35)	
4	3 (4.92)	315 (6.79)	
Missing	0 (0.00)	49 (1.06)	
mMRC Questionnaire (*n* (%))			
Grade 0–1	16 (26.23)	244 (5.26)	<0.0001
Grade ≥2	45 (73.77)	4395 (94.74)	
GOLD Group (*n* (%))			
A	0 (0.00)	0 (0.00)	<0.0001
B	16 (26.23)	2426 (52.30)	
C	16 (26.23)	244 (5.26)	
D	29 (47.54)	1969 (42.44)	
Patients with concomitant diseases (*n* (%))			
No	9 (14.75)	1568 (33.80)	0.0017
Yes	52 (85.25)	3071 (66.20)	
Patients with concomitant medication (*n* (%))			
No	15 (24.59)	2405 (51.84)	<0.0001
Yes	46 (75.41)	2234 (48.16)	
Smoking status (*n* (%))			
Smoker	31 (50.82)	2232 (48.11)	0.2659
Ex-Smoker	28 (45.90)	1973 (42.53)	
Non-Smoker	2 (3.28)	434 (9.36)	
Patients with exacerbations in the last 12 months prior to study (*n* (%))			
≥1	51 (83.61)	3321 (71.59)	0.0383
0	10 (16.39)	1318 (28.41)	
Number of exacerbations in the last 12 months prior to study			
Patients with available data (*n*)	61	4639	0.0023
Mean	1.70	1.20	
SD	1.43	1.13	
Min	0.00	0.00	
Median	1.00	1.00	
Max	8.00	12.00	

**Table 2:** Pearson correlation between the CCQ score and mMRC at baseline, and the number of exacerbations within the last 12 months.

		Switzerland		Other Countries	
		mMRC at Baseline	Number of Exacerbations within the Last 12 Months	mMRC at Baseline	Number of Exacerbations within the Last 12 Months
Change in CCQ-Score	Correlation coefficient r	−0.21	−0.10	−0.26	−0.19
	*p*-value	0.1068	0.4543	<0.0001	<0.0001
	*n*	61	61	4639	4639

## Data Availability

The authors confirm that the data supporting the findings of this study are available within the article. To ensure independent interpretation of clinical study results, Boehringer Ingelheim grants all external authors access to relevant material, including participant-level clinical study data, as needed by them to fulfill their role and obligations as authors under the ICMJE criteria. Clinical study documents and participant clinical study data are available to be shared on request from the corresponding author.
